# Design, Synthesis and Biological Evaluation of *Trypanosoma brucei* Trypanothione Synthetase Inhibitors

**DOI:** 10.1002/cmdc.201100420

**Published:** 2011-12-08

**Authors:** Daniel Spinks, Leah S Torrie, Stephen Thompson, Justin R Harrison, Julie A Frearson, Kevin D Read, Alan H Fairlamb, Paul G Wyatt, Ian H Gilbert

**Affiliations:** [a] Drug Discovery Unit, Division of Biological Chemistry & Drug Discovery, College of Life Sciences, University of DundeeDundee DD1 5EH (UK); Drug Discovery Unit, Division of Biological Chemistry & Drug Discovery, College of Life Sciences, University of DundeeDundee DD1 5EH (UK)

**Keywords:** antiprotozoal agents, drug design, *Trypanosoma brucei*, trypanothione synthetase

## Abstract

Trypanothione synthetase (TryS) is essential for the survival of the protozoan parasite *Trypanosoma brucei*, which causes human African trypanosomiasis. It is one of only a handful of chemically validated targets for *T. brucei* in vivo. To identify novel inhibitors of *Tb*TryS we screened our in-house diverse compound library that contains 62 000 compounds. This resulted in the identification of six novel hit series of *Tb*TryS inhibitors. Herein we describe the SAR exploration of these hit series, which gave rise to one common series with potency against the enzyme target. Cellular studies on these inhibitors confirmed on-target activity, and the compounds have proven to be very useful tools for further study of the trypanothione pathway in kinetoplastids.

## Introduction

Human African trypanosomiasis (HAT), or sleeping sickness, is endemic in sub-Saharan Africa, claiming the lives of about 30 000 people every year and putting approximately 60 million people at risk of infection.^1^ HAT is a progressive and fatal disease caused by the protozoan parasites *Trypanosoma brucei gambiense* and *T. b. rhodesiense*, which are transmitted to the human host by the bite of the tsetse fly. If left untreated the disease progresses to the central nervous system and is ultimately fatal. There is a clinical need for more effective drug therapies. Current therapies are toxic and have inappropriate treatment regimens for a rural African setting. There are also problems with treatment failures.^1^–^3^

Differences in metabolic pathways have been discovered between parasite and host, which may be exploited for drug discovery programmes. An example of such a difference is found in thiol metabolism and the response of *T. brucei* to oxidative stress.^4^–^8^ Studies have shown that trypanosomatid parasites are uniquely dependent on trypanothione (*N*^1^,*N*^8^-bis(glutathionyl)spermidine) as their principal thiol, in contrast to most other organisms (including their mammalian hosts) that use glutathione (γ-l-glutamyl-l-cysteinylglycine, GSH).^9^ In *T. brucei* trypanothione is synthesised from GSH and spermidine (Spd) by an ATP-dependent C–N ligase, trypanothione synthetase (TryS; EC 6.3.1.9), with *N*^1^- and *N*^8^-glutathionylspermidine as intermediates.^10^, ^11^

Selective inhibition of the trypanothione pathway with chemical agents (targeting trypanothione reductase, tryparedoxin, and tryparedoxin peroxidise) or classical gene knockout studies have shown a clear trypanocidal effect.^12^, ^13^ *Tb*TryS has also been genetically validated as a drug target, with RNAi and gene knockout studies confirming that *Tb*TryS is essential for *T. brucei* growth in both bloodstream and procyclic forms, and that there is no alternative bypass mechanism available to the parasite.^14^, ^15^

Before commencing a drug discovery programme, *Tb*TryS was assessed for its suitability as a drug target using the traffic light scoring system that we have developed in house.^16^ The assessment indicated *Tb*TryS is an attractive target for drug development, especially as it is unlikely to have resistance or toxicity issues, as there is no obvious bypass metabolism or equivalent enzyme in humans.^10^ The main concern was the potential druggability of the target. Because the active site of *Tb*TryS is large enough to accommodate trypanothione and precursors, this may be an issue if the active site is a large featureless pocket, as is observed in *T. brucei* trypanothione reductase (*Tb*TryR).^17^ However, the structure of TryS from *Leishmania major* suggests this is not the case,^18^ and the potential to co-crystallise ligands with the protein to inform a chemistry programme was a distinct advantage. Importantly, *Tb*TryS is a bifunctional enzyme, which catalyzes the biosynthesis and hydrolysis of the GSH–Spd adduct trypanothione. The two catalytic domains are separate in *Leishmania*. The N-terminal domain is a cysteine-containing amidohydrolase/peptidase amidase site, with the C-terminal ATP grasp domain responsible for the synthetase activity of the enzyme.^18^

**Figure 1:**
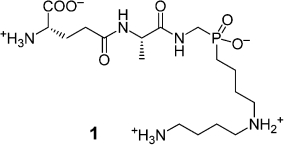
Previously published *Tb*TryS inhibitor: compound **1**, *K*_i_^app^: 500–1200 nm.

Figure 1 shows the only previously disclosed inhibitor of *Tb*TryS, compound **1**.^19^ Whilst a valuable tool molecule, the optimisation and development of this phosphinate inhibitor into a potential clinical candidate is limited due to the peptidic nature of such a compound, with a high polar surface area (PSA), and charges at physiological pH (which are detrimental for cellular penetration, metabolic stability, bioavailability, and blood–brain barrier permeability).

Herein we describe a medicinal chemistry programme to develop inhibitors of *Tb*TryS, which gave rise to some potent compounds. We recently reported the biological experiments to chemically validate *Tb*TryS using five of these compounds: **9**, **20**, **71**, **84**, and **89**.^15^, ^20^

## Results and Discussions

### High-throughput screening

As previously described, a high-throughput screening assay for *Tb*TryS was developed and validated.^20^ *Tb*TryS was then screened against a 62 000 compound diversity set at single-point concentration (30 μm). This gave rise to >720 hits (compounds with *Tb*TryS percentage inhibition >33 % at 30 μm). These hits were clustered and filtered down to 174 compounds that underwent potency testing (10-point, half-log dilution dose–response curves from which an accurate IC_50_ could be calculated). This gave rise to the six putative hit series, plus a number of singletons. Appropriate hits were then re-purchased to confirm identity and activity. A round of purchasing (where available) and synthesis of analogues was initiated to validate the series and to investigate the SAR around these potential hit series to assess optimisation towards lead series.

Table 1 shows the hit series identified from the HTS screen, and the ligand efficiencies of compounds from these series.^21^ Although there are six distinct chemical series shown in Table 1, the series can be clustered into two distinct groups that share common pharmacophoric features. Attempts were made to co-crystallise hit ligands in the protein to obtain an X-ray crystal structure showing ligands in the binding domain, but unfortunately these have been unsuccessful so far.

**Table 1:** Hit series clusters identified from HTS.

Series	Structure	LE[a]
**1 a**	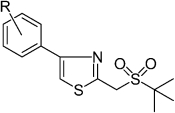	0.44
**1 b**	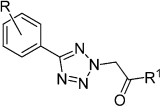	0.45
**1 c**	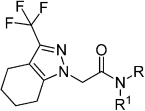	0.33
**2 a**	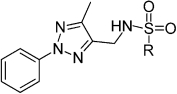	0.33
**2 b**	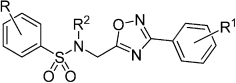	0.30
**2 c**	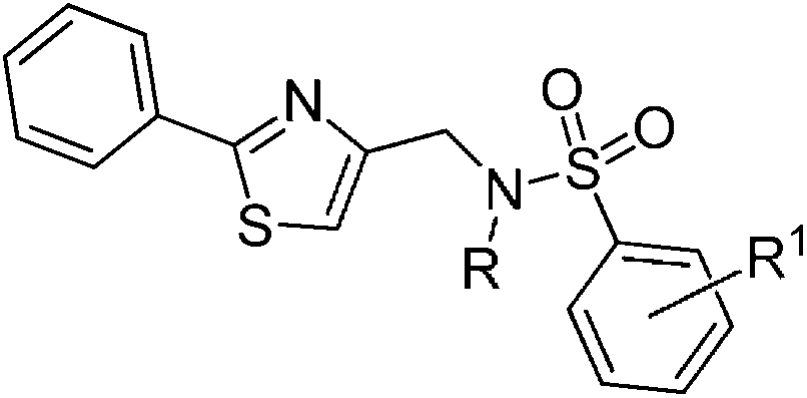	0.29

[a] Ligand efficiency, calculated as −0.6×ln(IC_50_)/(heavy atom count).^21^

### Initial hit exploration around series/group 1

Group 1 series were identified as having a common pharmacophore with two hydrogen bond acceptors (HBA) in 1,5 relationship, with one of the HBAs from a heterocyclic ring. The pharmacophore also includes regions of hydrophobicity, indicating a potential lipophilic pocket.

#### Hit series 1 a: thiazole methylene sulfone

The synthetic route to prepare this series involved the condensation of a thioamide with an appropriate α-bromoketone (Table 2), based on the methodology of Dunn et al.^22^ The corresponding α-bromoketones could be bought or synthesised from the corresponding acetyl compound (see Experimental Section below for more details). Activities of hit series **1 a** compounds against *Tb*TryS are listed in Table 2.

**Table 2:** SAR around hit series 1 a.

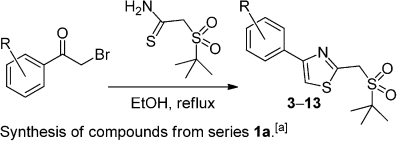					
Compd	R	IC_50_ [μm]^[b]^	Compd	R	IC_50_ [μm]^[b]^
**3**	H	1.2	**9**	3-F	0.4
**4**	2-CH_3_	3.2	**10**	4-CH_3_	>100
**5**	2-F	1.1	**11**	4-CF_3_	>100
**6**	3-OCH_3_	12.0	**12**	3,4-di-Cl	2.5
**7**	3-CF_3_	1.1	**13**	3,4-di-F	0.4
**8**	3-Cl	0.4			

[a] Yields: 60–87 %; the synthesis of compound **9** was previously described by Torrie et al.^20^ [b] IC_50_ values against *Tb*TryS.

Exploration around the scaffold of **1 a** (Table 2) shows that 2-substitution with a small substituent, methyl (compound **4**) and fluorine (compound **5**), is tolerated without loss of potency. An electron-donating substituent at the 3-position, as in compound **6**, gave a 10-fold loss in potency relative to unsubstituted compound **3**; however, all potency was regained if the 3-position group was changed to an electron-withdrawing group, such as trifluoromethyl (compound **7**). In addition, a slight improvement in potency was observed for the 3-F (**9**) and 3-Cl (**8**) derivatives (IC_50_: 0.4 μm). These analogues showed sub-micromolar potency is achievable within this hit series. Substitution at the 4-position was not tolerated with either an electron-withdrawing or -donating group. Both methyl (**10**) and trifluoromethyl (**11**) were found to be inactive. However, 3,4-disubstitution with an electron-withdrawing substituent at the 3-position restored potency (compounds **12** and **13**), although ligand efficiency was decreased.

#### Hit series 1 b: tetrazole methylene carbonyl

In this series, the thiazole subunit is replaced by a tetrazole, and the sulfone by a carbonyl group. The tetrazoles were alkylated with α-bromoketones (Table 3). Amide analogue **23** was synthesised via an amide coupling of the corresponding acid, which in turn was obtained from saponification of the ethyl ester.

**Table 3:** SAR around hit series 1 b.

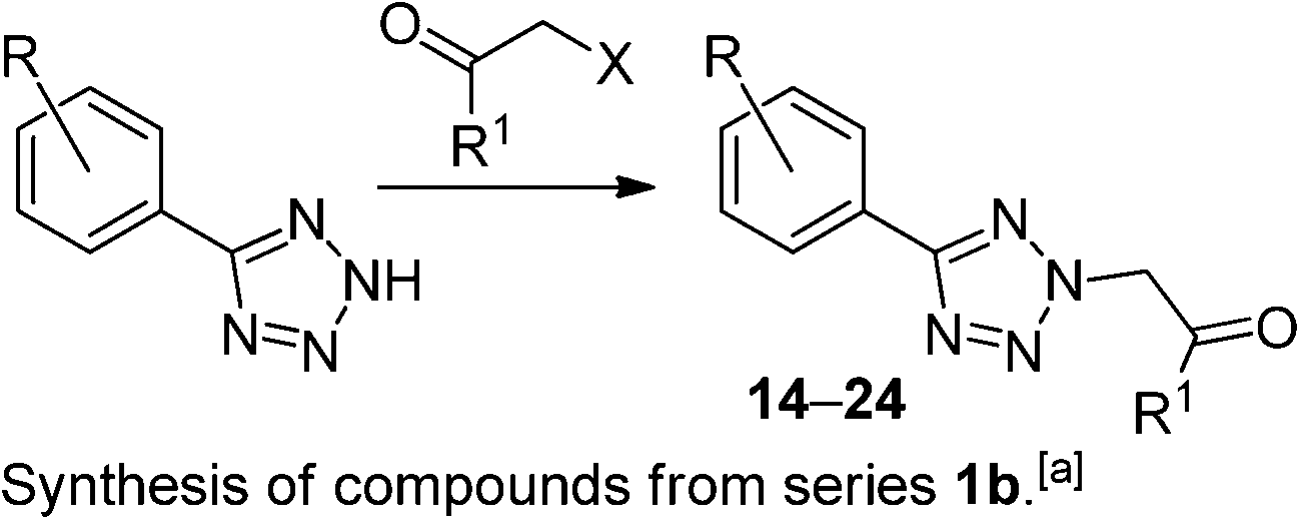			
Compd	R	R^1^	*Tb*TryS IC_50_ [μm]
**14**	H	*t*Bu	8.8
**15**	4-CF_3_	*t*Bu	>100
**16**	3-Cl, 4-CH_3_	*t*Bu	7.2
**17**	3-F	*t*Bu	1.5
**18**	3-CF_3_	*t*Bu	3.5
**19**	3,5-di-F	*t*Bu	1.9
**20**	3,5-di-Cl	*t*Bu	0.3
**21**	3,5-di-Cl	CH_3_	6.5
**22**	3,5-di-Cl	CH_2_CH_3_	2.7
**23**	3,5-di-Cl	N(CH_3_)_2_	0.6
**24**	3,5-di-F	Ph	45

[a] Conditions: NaI, K_2_CO_3_, DMF, 90 °C; yields: 44–86 %; the synthesis of compound **20** was previously described by Torrie et al.^20^

Subtle trends in SAR similar to series **1 a** were observed in series **1 b** (Table 3). In particular, there was a 25-fold gain in potency for the 3,5-dichloro analogue **20**, relative to the unsubstituted scaffold **14**. This could be due to the greater lipophilicity of this compound causing an increase in nonspecific binding, although the ligand efficiency improved (LE from 0.39 to 0.45), suggesting a favourable specific interaction. It was possible to modify the ketone moiety to an amide without loss of potency (compare amide **23** (IC_50_=0.6 μm), with ketone **20** (IC_50_=0.3 μm)). In going from methyl (**21**) to ethyl (**22**) to *tert*-butyl ketone (**20**), an improvement in potency was observed (IC_50_: 8.8 to 6.5 to 0.3 μm, respectively), indicating lipophilic bulk in this area is required. In contrast, the phenyl ketone **24** shows a marked loss in potency, with an IC_50_ value of 45 μm.

#### Hit series 1 c: tetrahydroindazole methylene amide

The pyrazole core for series **1 c** was made by condensation of ethyl hydrazinoacetate with the commercially available dione. The ester was then hydrolysed, allowing amide couplings using standard methodology (Table 4).^23^, ^24^

**Table 4:** SAR around hit series 1 c.

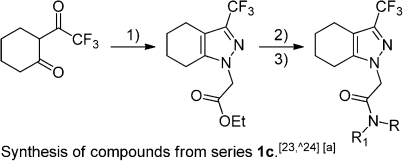			
Compd	R^1^	R	*Tb*TryS IC_50_ [μm]
**25**	H	(+/−)-CH(CH_3_)Ph	5.6
**26**	H	CH_2_-4-chlorophenyl	3.0
**27**	H	CH_2_-2-thiophene	3.9
**28**	H	CH_2_CH_2_Ph	5.6
**29**	H	(+/−)-CH(CH_3_)CH_2_CH_3_	12
**30**	H	CH_2_CH_2_CH_3_	13
**31**	H	CH_2_CH_2_OCH_3_	30
**32**	H	Ph	>100
**33**	H	*c*Hex	>100
**34**	CH_2_Ph	CH_2_Ph	>100
**35**	CH_2_CH_3_	CH_2_CH_3_	15

[a] Conditions: 1) ethyl 2-hydrazinylacetate, Et_3_N, EtOH, reflux, 61 % yield; 2) 2 m NaOH, THF/H_2_O (2:1), 89 % yield; 3) RR^1^NH, EDC⋅HCl, HOBt, DIPEA, DMF, 50 °C; yields: 79–80 %.

Small changes to optimise the amide group in this series generated a “flat” SAR plateau (**25**–**28**; Table 4). Aliphatic amides (**29**–**31**) were tolerated, albeit at weaker potency, but bulky rigid substitution with the phenyl **32** and cyclohexyl **33** moieties was not tolerated, with complete loss in potency observed. Tertiary amide **34** with two bulky benzyl groups also lost all potency, but the less bulky diethyl amide **35** retained some inhibitory activity.

### Initial hit exploration around series/group 2

Following the HTS campaign we also identified three hit series based around a second pharmacophore. This second group of hit series described a slightly different pharmacophore, with the heteroatom of the heterocycle in a 1,6 relationship with the HBA motif of the sulfonamide. These hit series had higher molecular weight and generally lower potency, with lower ligand efficiency (see Table 1). To explore the SAR around these series, compounds were purchased and screened. Data for these are listed in Tables 5, 6, and 7.

**Table 5:** SAR around hit series 2 a.

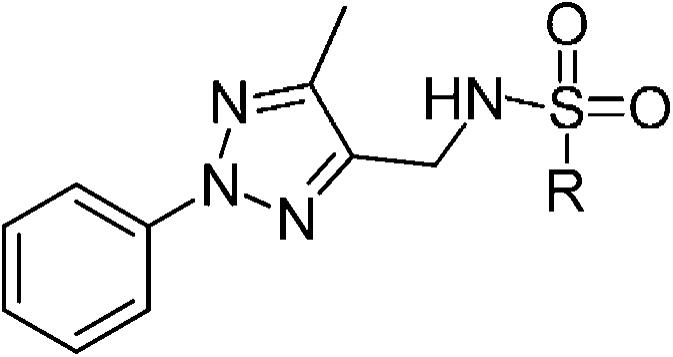		
Compd	R	*Tb*TryS IC_50_ [μm]
**36**	2-thiophene	8.5
**37**	5-(2,4-dimethylthiazole)	2.2
**38**	4-(1,3,5-trimethylpyrazole)	2.6
**39**	1-naphthyl	>100
**40**	2,5-dimethoxyphenyl	>100
**41**	3-(2,5-dimethylthiophene)	24
**42**	*t*Bu	37

**Table 6:** SAR around hit series 2 b.

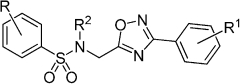				
Compd	R^2^	R^1^	R	*Tb*TryS IC_50_ [μm]
**43**	H	H	H	>50
**44**	H	2-CH_3_	H	15
**45**	H	2-Cl	H	25
**46**	H	H	2,5-di-Cl	16
**47**	H	H	4-NHCOCH_3_	5.9
**48**	H	H	4-OCH_3_	11
**49**	H	2-CH_3_	4-OCH_3_	4.0
**50**	H	2-CH_3_	4-NHCOCH_3_	1.7
**51**	H	2-Cl	4-NHCOCH_3_	3.7
**52**	CH_3_	H	4-NHCOCH_3_	>50

**Table 7:** SAR around hit series 2 c.

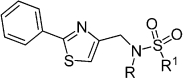			
Compd	R	R^1^	*Tb*TryS IC_50_ [μm]
**53**	H	2,5-difluorophenyl	6.1
**54**	H	phenyl-4-COCH_3_	8.7
**55**	H	2,5-dichlorophenyl	6.3
**56**	H	3,4-dichlorophenyl	5.8
**57**	H	2-naphthyl	2.4
**58**	Ph	4-methylphenyl	>100

None of the group 2 series offered any benefits in terms of potency over the compound series from group 1. In addition, the DMPK data indicated less favourable properties of group 2 series compounds, relative to data for compounds from group 1 (see Table 12 below). Therefore, further optimisation work was focussed on the group 1 series.

### Hit to lead optimisation strategy

**Figure 2:**
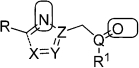
Group 1 common pharmacophore: R and R^1^ are hydrophobic binding domains; N and O (boxed) are the hydrogen bond acceptor motifs (1,5 relationship); X, Y, and Z are C, N, O, or S; Q is C or SO (sulfone).

**Figure 3:**
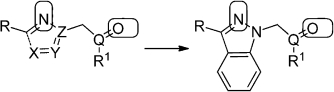
Generation of the new scaffold.

Hit series **1 a**–**c** were successfully validated and shared an overlapping pharmacophore for *Tb*TryS activity (Figure 2), demonstrating clear SAR and a visible potential for further optimisation. The pharmacophoric features of the three group 1 series scaffolds were hybridised into one new core scaffold, which was based on an indazole (Figure 3). One of the indazole nitrogen atoms becomes the HBA, and the other is used for attachment of the other HBA. This indazole series is also predicted to have reasonable physicochemical properties: low molecular weight, clog*P*<5, and low PSA. For example, compound **60** (Table 8) has *M*_r_=292 Da, clog*P*=4.0, and PSA=35 Å^2^.

**Table 8:** SAR around lead series 3.

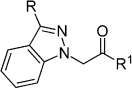				
Compd	R	R^1^	*Tb*TryS IC_50_ [μm]	LE^[a]^
**59**	Ph	OCH_3_	20	0.32
**60**	Ph	*t*Bu	0.15	0.43
**61**	4-chlorophenyl	*t*Bu	>100	
**62**	4-indole	*t*Bu	3.7	0.30
**63**	4-(*N*-methyl)indole	*t*Bu	>100	
**64**	2-naphthyl	*t*Bu	>100	
**65**	4-pyridyl	*t*Bu	0.3	0.41
**66**	2-furanyl	*t*Bu	1.9	0.38
**67**	3-pyridyl	*t*Bu	3.0	0.35
**68**	3-thiophenyl	*t*Bu	0.4	0.42
**69**	4-(*N*-isobutyl)pyrazole	*t*Bu	>100	
**70**	3-chlorophenyl	*t*Bu	0.35	0.39
**71**^[b]^	3-fluorophenyl	*t*Bu	0.09	0.42

[a] Ligand efficiency, calculated as −0.6×ln(IC_50_)/(heavy atom count).^21^ [b] Compound **71** reported previously.^20^

### Validation of the indazole series

**Scheme 1:**
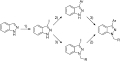
General synthesis of indazole analogues. Conditions: 1) KOH, I_2_, DMF, as in Edwards et al.,^25^ 89 % yield; 2) ArB(OH)_2_, Na_2_CO_3_, DME, EtOH, H_2_O, yields: 50–78 %; 3) NaH, DMF, α-haloketone (or other), yields: 37–62 %. Synthesis of compounds **71** and **89** were described previously.^20^

The indazole scaffold was prepared as shown in Scheme 1. Indazole was first iodinated (at the 3-position) using standard conditions.^25^ A Suzuki reaction afforded the 3-aryl intermediate. Alkylation of the N1 nitrogen atom with the required α-chloroketone (or arylmethylchloride) gave final products **60**, **71**, and **75**–**79**. Alternatively for compounds **61**–**70**, the alkylation of the 3-iodoindazole was completed first and the Suzuki reaction, to add the aryl substituent, was employed as a final step.

### 3-Indazole substitution

Table 8 shows the data for key compounds from the initial SAR study, describing changes to the aromatic group at position 3 of the indazole (depicted R in the structure). The initial compound **60** (R=phenyl), had an IC_50_ value of 150 nm in the *Tb*TryS enzyme assay (LE calculated as 0.43) and was the most potent compound to date. *Para* substituents led to loss in activity (e.g. **61**, **63**, **64**, and **69**). This is possible evidence for the presence of a tight binding pocket into which the aromatic subunit sits. A variety of heterocycles were tolerated, although some gave a 10-fold loss in activity, such as the 2-furanyl compound **66** and 3-pyridyl compound **67**. The 3-fluorophenyl motif (**71**, IC_50_=90 nm, LE=0.42) was more potent than 3-chlorophenyl (**70**, IC_50_=354 nm), and was used as the standard template for exploration of substituent SAR around the R^1^ position (see Tables 9–11 below).

Having established the indazole as a potent heterocyclic core with good ligand efficiency, a number of SAR studies were carried out to explore chemical optimisation around this scaffold.

### Investigation of alternative HBA moieties

We were concerned that the ketone group could react with nucleophiles, so a number of alternative HBAs were investigated. The initial sulfone analogue **74** (*Tb*TryS IC_50_=120 nm) was found to be equipotent to the ketone **60** (Table 9). The position from which the pendant HBA is attached to the core scaffold was also investigated. In compounds **72** and **73** the sulfone HBA is appended from the 3-position of the indazole core (Table 9). This modification had little effect on activity, which is probably due to the symmetry of the molecule. For ease of synthesis, further HBA modifications were investigated at the N1 position of the indazole.

**Table 9:** SAR around indazole sulfone analogues.

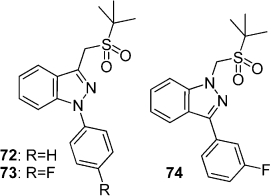			
Compd	R	*Tb*TryS IC_50_ [μm]	LE^[a]^
**72**	H	0.22	0.40
**73**	4-fluoro	0.08	0.41
**74**	–	0.12	0.40

[a] Ligand efficiency, calculated as −0.6×ln(IC_50_)/(heavy atom count).^21^

Approaches were made to use a heterocycle as the second HBA motif (Table 10). Pyridine and oxazole were investigated, but showed lower activity than the ketone or sulfone groups.

**Table 10:** SAR around pendant heterocyclic side chain indazoles.

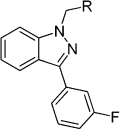			
Compd	R	*Tb*TryS IC_50_ [μm]	LE^[a]^
**74**	SO_2_*t*Bu	0.12	0.40
**75**	2-pyridyl	2.9	0.33
**76**	3-pyridyl	4.5	0.32
**77**	2,5-dimethyl-4-oxazole	28	0.26
**78**	5-phenyl-3-isoxazole	>100	
**79**	5-methyl-3-isoxazole	>100	

[a] Ligand efficiency, calculated as −0.6×ln(IC_50_)/(heavy atom count).^21^

#### Amide HBA analogues

**Scheme 2:**
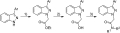
Synthesis of amide analogues. Conditions: 1) NaH, DMF, ethyl bromoacetate, as in Fujimura et al.,^29^ yields: 37–62 %; 2) NaOH, THF/H_2_O, 95 % yield; 3) HOBt, EDC, DMF, DIPEA, R_1_R_2_NH; yields: 40–48 %.

Alkylation of the 3-arylindazole (synthesis shown in Scheme 2) with ethylbromoacetate, followed by saponification, yielded the indazole-*N*-acetic acid intermediate. This was coupled under standard amide coupling conditions to the appropriate amine to give access to amide analogues.

Encouragingly, the ketone subunit could be replaced by a simple amide without loss of potency or ligand efficiency (Table 11). Thus the diethyl and dimethyl amides (**87** and **88**) had IC_50_ values similar to that of the ketone **71**, with compound **88** having a ligand efficiency of 0.44. Potency was retained when fusing the dialkyl amide into an *N*-piperidine amide (**89** *Tb*TryS IC_50_=135 nm), but activity was completely abolished with a larger alkyl aromatic substituent (e.g. **86** IC_50_>100 μm).

**Table 11:** Amide side chain indazoles.

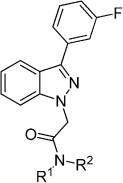				
Compd	R^1^	R^2^	*Tb*TryS IC_50_ [μm]	LE^[a]^
**80**	(CH_2_)_3_-*N*-methylpiperazine	H	0.83	0.28
**81**	(CH_2_)_3_-*N*1-imidazole	H	0.35	0.32
**82**	(CH_2_)_2_-*N*-Boc-piperazine	H	2.9	0.22
**83**	(CH_2_)_2_N(CH_3_)_2_	H	0.145	0.38
**84**^[b]^	(CH_2_)_3_N(CH_3_)_2_	H	0.045	0.39
**85**	(CH_2_)_3_N(CH_3_)_2_	CH_3_	0.17	0.35
**86**	CH_2_-4-chlorophenyl	H	>100	
**87**	CH_2_CH_3_	CH_2_CH_3_	0.20	0.39
**88**	CH_3_	CH_3_	0.11	0.44
**89**^[b]^	fused into piperidine ring		0.14	0.38
**90**	(CH_2_)_2_-*N*-methylpiperazine	H	0.86	0.29

[a] Ligand efficiency, calculated as −0.6×ln(IC_50_)/(heavy atom count).^21^ [b] Compounds **84** and **89** reported previously.^15^, ^20^

Addition of an appended basic amine, to potentially pick up the *Tb*TryS endogenous substrate Spd binding domain interactions and improve ligand potency, was investigated. This basic group could also improve the aqueous solubility of our compounds and lower the lipophilicity (log*P*). Although the piperazine-containing compounds **90** and **80** lost potency (ninefold relative to **71**), and were less efficient binders (ligand efficiencies of 0.29 and 0.28 respectively), they were still sub-micromolar inhibitors of the *Tb*TryS enzyme, with *Tb*TryS IC_50_ values of 0.86 and 0.83 μm, respectively. If the second basic amine centre was removed and the compounds were truncated to make the C2- and C3-linked dimethyl amine compounds **83** and **84**, a significant improvement in potency (six- and 18-fold, respectively) over the analogous piperazines was observed. These compounds were also significantly more efficient binders than compounds **80** and **90**, with respective ligand efficiencies of 0.38 and 0.39. The C3-linked dimethylamine compound **84** was observed to be the most potent compound to date, with a *Tb*TryS IC_50_ value of 45 nm. Compound **84** shows improved physicochemical properties over compound **60**, especially in decreased lipophilicity, with a clog*P* value of 2.8 (1.3 units lower than that of **60**), and *M*_r_=354 Da and PSA=50 Å^2^.

As compound **84** shows similar potency to an analogue not containing an appended amine (**71**, IC_50_ 90 nm) it is unlikely that compound **84** has picked up the Spd binding domain. This conclusion is supported by competition binding studies which revealed that the compounds displayed mixed inhibition with respect to Spd, and did not show classical competitive binding kinetics.^20^ Finally, capping the NH group of the amide of **84** with a methyl group (compound **85**) resulted in a fourfold decrease in potency, and the C3-linked imidazole **81** was eightfold less potent than the dimethylamine.

### Cell potency and DMPK parameters for key compounds

Following the discovery of promising novel TryS inhibitors, compounds were progressed into a trypanosomal cell proliferation assay and a human cell counter-screen. Selected compounds were also screened in an in vitro metabolic clearance assay (Table 12), to assess suitability for series progression. Metabolic stability studies using pooled human liver microsomes indicate a range of stabilities. Compounds from the group 2 series are highly metabolically unstable. However, compounds **8**, **26**, and **71** are reasonably metabolically stable, suggesting nothing fundamentally problematic with these scaffolds in terms of cytochrome P450-driven metabolism.

Table 12 also shows cell data for key compounds from several of the various series of *Tb*TryS inhibitors discovered. Although these compounds were not toxic to the MRC5 mammalian cell line, there was up to a 100-fold decrease in going from enzyme to trypanosomal cell efficacy, even with the lead compounds **71** and **84** (*Tb*TryS IC_50_: 90 and 45 nm, respectively). While these cell potencies are equivalent to the drugs eflornithine (22 μm) and nifurtimox (2 μm) currently in clinical use for late-stage human African trypanosomiasis, they are much less potent than the alternative arsenic-containing drug melarsoprol (8 nm), although arsenic-based compounds do show significant toxicity in the clinic.^26^

**Table 12:** Trypanosome cell, human (MRC5) cell, and pooled human microsomal intrinsic clearance (*Cl*_i_) data for key (representative) compounds from series 1–3.

Compd	Series	*Tb*TryS IC_50_ [μm]	*Tb* EC_50_ [μm]	MRC5 EC_50_ [μm]	*Cl*_i_ [mL min^−1^ g^−1^]
**8**	**1 a**	0.41	22	>50	0.9
**26**	**1 c**	14	14	>50	3.0
**36**	**2 a**	8.5	32	>50	9.1
**57**	**2 c**	2.4	19	>50	17
**71**	**3**	0.09	7.9	>50	1.6
**84**	**3**	0.045	5.4	20	ND^[a]^
**89**	**3**	0.14	5.1	>50	ND^[a]^

[a] Not determined.

As this large potency shift between the enzyme IC_50_ values and parasite EC_50_ values was unexpected, further experiments were carried out to confirm whether hit compounds were entering the cell and acting on-target. As described fully elsewhere, exposing *T. brucei* parasites to the model *Tb*TryS inhibitors **89** and **84** (2×EC_50_ for 72 h) resulted in trypanothione levels dropping to <10 % of wild-type levels.^15^, ^20^ In addition, there was a corresponding increase in the *Tb*TryS substrate GSH, providing strong evidence that these compounds were acting on-target.

As previously reported, the on-target effects of these hit compounds were further confirmed by generating *Tb*TryS single knockout (SKO) and *Tb*TryS overexpressing (OE) cell lines. Western blot analysis and densitometry demonstrated that *Tb*TryS protein levels were decreased in the SKO cells and elevated in the OE cell line, relative to wild-type cells, and these cell lines showed the expected changes in potency to **89** (EC_50_ values: 20.4, 6.9, and 44.5 μm for wild-type, SKO, and OE cell lines, respectively) and **84** (EC_50_ values: 7.1, 1.2, and 23.3 μm for wild-type, SKO, and OE cell lines, respectively), confirming that *Tb*TryS is the specific target of these compounds.^15^, ^20^

## Conclusions

In this work we successfully took HTS hits, clustered them into putative hit series, and rationalised their activities based on common pharmacophores. Initial investigation of SAR around the hit series confirmed an overlapping pharmacophore, and the optimisation potential of group 1 hit series in particular. Following the SAR on group 1 series, a hybridisation strategy and scaffold-hopping approach led us to discover the indazole lead series. Optimisation of this series for potency and improved DMPK properties led to compounds **71** and **84**, which displayed in vitro enzyme potencies >10-fold improved over the best HTS hits. Attempts so far to co-crystallise our inhibitors with the *Tb*TryS enzyme have failed to produce robust data.

Although these indazoles inhibit *Tb*TryS with IC_50_ values of <100 nm, they failed to show sub-micromolar potency in a trypanosome proliferation assay. This can be rationalised by the observation that parasites can survive with low levels of trypanothione beyond the timeframe of the standard whole-parasite proliferation assay. The extension of the time-course in screening assay format is prohibited by the need for repeated dilutions of samples to remain in log-phase growth, leading to unacceptable variability. The lead compounds do, however, show a robust biochemical effect in *T. brucei*, and are proven to act on-target, inhibiting *Tb*TryS in cells.^15^, ^20^ The current lead compounds could also prove very useful in combination therapy with known trypanocides (such as melarsoprol), as studies have revealed TryS-depleted *T. brucei* procyclics are significantly more susceptible to trypanocides.^27^ Our compounds are the most advanced, potent, and drug-like (as predicted by physicochemical and in vitro DMPK properties) inhibitors of *Tb*TryS reported to date, and are extremely useful leads to further explore the trypanothione pathway in kinetoplastids.

## Experimental section

### Chemistry

^1^H NMR spectra were recorded on either Bruker Avance DPX 500 or Bruker Avance 300 spectrometers. Chemical shifts (*δ*) are expressed in ppm. Signal splitting patterns are described as singlet (s), broad singlet (bs), doublet (d), triplet (t), quartet (q), multiplet (m) or combination thereof. LC–MS analyses were performed with either an Agilent HPLC 1100 series instrument connected to a Bruker Daltonics MicrOTOF, or an Agilent Technologies 1200 series HPLC connected to an Agilent Technologies 6130 quadrupole LC–MS; both instruments were connected to an Agilent diode-array detector. LC–MS chromatographic separations were conducted with a Phenomenex Gemini C_18_ column, 50×3.0 mm, 5 μm particle size; mobile phase, H_2_O/CH_3_CN +0.1 % HCOOH 80:20→5:95 over 3.5 min, and then held for 1.5 min; flow rate: 0.5 mL min^−1^. High-resolution electrospray MS measurements were performed on a Bruker Daltonics MicrOTOF mass spectrometer. Thin-layer chromatography (TLC) was carried out on Merck silica gel 60 F_254_ plates using UV light and/or KMnO_4_ for visualisation. TLC data are given as the *R*_f_ value with the corresponding eluent system specified in brackets. Column chromatography was performed using RediSep® 4 or 12 g silica pre-packed columns. LCMS chromatographic separations were conducted with a Waters Xbridge C_18_ column, 50 mm × 2.1 mm, 3.5 μm particle size; Method A: mobile phase, H_2_O/CH_3_CN + 0.1 % NH_3_; linear gradient 80:20→5:95 over 3.5 min, and then held for 1.5 min; flow rate 0.5 mL min^−1^. All reactions were carried out under dry and inert conditions, unless otherwise stated.

### Compounds in series 1 a:

**2-(*tert*-Butylsulfonylmethyl)-4-(3-fluorophenyl)thiazole (9)**: Synthesis previously described.^20^ To a stirred solution of 3-fluoroacetophenone (250 mg, 1.81 mmol) in THF (6 mL), was added trimethylphenylammonium tribromide (681 mg, 1.81 mmol) solution in THF (4 mL). The reaction was stirred at room temperature for 18 h; the resulting white precipitate was filtered off, and the filtrate was added to petroleum ether (PE; 20 mL). The PE solution containing the product was washed with H_2_O (30 mL) and then dried (MgSO_4_). The solvent was then removed in vacuo to give intermediate **2-bromo-1-(3-fluorophenyl)ethanone** (390 mg, 99 %) as a pale-yellow oil; [*M*+H]^+^=217/219. To a stirred solution of 2-bromo-1-(3-fluorophenyl)ethanone (326 mg, 1.8 mmol) in EtOH (20 mL) was added 2-(*tert*-butylsulfonyl)ethanethioamide (386 mg, 1.98 mmol), and the reaction was heated at reflux and stirred for 2 h. The solvent was then removed in vacuo to give a crude residue which was purified by column chromatography (CH_2_Cl_2_→10 % MeOH/CH_2_Cl_2_ eluent) to give the title compound **9** (380 mg, 81 %) as a white solid: ^1^H NMR ([D_6_]DMSO, 500 MHz): *δ*=8.40 (1 H, bs), 8.31 (2 H, m), 7.70 (2 H, m), 5.15 (2 H, bs), 1.4 (9 H, bs); [*M*+H]^+^=314; HRMS C_14_H_16_FNO_2_S_2_ calcd: 314.0679, obsd: 314.0690.

Compounds **3**–**8** were prepared according to the same procedures as described above for the preparation of compound **9**, using the corresponding acetophenone.

**2-((*tert*-Butylsulfonyl)methyl)-4-(phenyl)thiazole (3)**: ^1^H NMR ([D_6_]DMSO, 500 MHz): *δ*=8.23 (1 H, s), 7.98 (2 H, dd, *J*=1.4, 8.5 Hz), 7.46 (2 H, t, *J*=7.5 Hz), 7.37 (1 H, t, *J*=12.5 Hz), 5.07 (2 H, bs), 1.39 ppm (9 H, bs); [*M*+H]^+^=296; HRMS C_14_H_17_NO_2_S_2_ calcd: 296.0767, obsd: 296.0773.

**2-((*tert*-Butylsulfonyl)methyl)-4-(2-methylphenyl)thiazole (4)**: ^1^H NMR ([D_6_]DMSO, 500 MHz): *δ*=7.88 (1 H, m), 7.59 (1 H, m), 7.31 (3 H, m), 5.06 (2 H, bs), 2.42 (3 H, s), 1.38 ppm (3 H, s); [*M*+H]^+^=310; HRMS C_15_H_19_NO_2_S_2_ calcd: 310.0930, obsd: 310.0943.

**2-((*tert*-Butylsulfonyl)methyl)-4-(2-fluorophenyl)thiazole (5)**: ^1^H NMR ([D_6_]DMSO, 500 MHz): *δ*=8.10 (2 H, m), 7.44 (1 H, m), 7.35 (2 H, m), 5.10 (2 H, m), 1.39 ppm (9 H, s); [*M*+H]^+^=314; HRMS C_14_H_16_FNO_2_S_2_ calcd: 314.0679, obsd: 314.0690.

**2-((*tert*-Butylsulfonyl)methyl)-4-(3-methoxyphenyl)thiazole (6)**: ^1^H NMR ([D_6_]DMSO, 500 MHz): *δ*=8.26 (1 H, s), 7.55 (1 H, m), 7.52 (1 H, m), 7.38 (1 H, t, *J*=8 Hz), 6.95 (1 H, m), 5.08 (2 H, s), 3.82 (3 H, s), 1.39 ppm (9 H, s); [*M*+H]^+^=326; HRMS C_15_H_19_NO_3_S_2_ calcd: 326.0879, obsd: 326.0888.

**2-(*tert*-Butylsulfonylmethyl)-4-(3-trifluoromethylphenyl)thiazole (7)**: ^1^H NMR ([D_6_]DMSO, 500 MHz): *δ*=8.49 (1 H, bs), 8.30 (2 H, m), 7.74 (2 H, m), 5.12 (2 H, bs), 1.4 ppm (9 H, bs); [*M*+H]^+^=364; HRMS C_15_H_16_F_3_NO_2_S_2_ calcd: 364.0647, obsd: 364.0639.

**2-((*tert*-Butylsulfonyl)methyl)-4-(3-chlorophenyl)thiazole (8)**: ^1^H NMR ([D_6_]DMSO, 500 MHz): *δ*=8.38 (1 H, bs), 8.03 (1 H, m), 7.95 (1 H, m), 7.51 (1 H, m), 7.44 (1 H, m), 5.09 (2 H, bs), 1.39 (9 H, s); ^13^C NMR (125 MHz, CDCl_3_): *δ*=23.8, 52.3, 61.6, 116.9, 124.4, 126.6, 128.4, 130.1, 134.9, 135.6, 154.0, 156.9 ppm; [*M*+H]^+^=330/332; HRMS C_14_H_16_ClNO_2_S_2_ calcd: 330.0384, obsd: 330.0384.

Compounds **9**–**13** were obtained from Maybridge.

### Compounds in series 1 b:

**1-(5-(3,5-Dichlorophenyl)-2*H*-tetrazol-2-yl)-3,3-dimethylbutan-2-one (20)**: Synthesis previously described.^20^ A mixture of commercially available 5-(3,5-dichlorophenyl)tetrazole (0.215 g, 1.0 mmol), 1-bromopinacolone (0.14 mL, 1.0 mmol) and diisopropylethylamine (0.19 mL, 1.1 mmol) in CH_3_CN (5 mL) was heated at 75 °C for 3 days. The mixture was cooled, diluted with EtOAc (15 mL), and filtered through a plug of silica. The filtrate was concentrated and purified by chromatography on silica (eluent: EtOAc/hexane 0→50 %) to give the title compound **20** (0.270 g, 86 %) as a white solid: ^1^H NMR (CDCl_3_, 300 K): *δ*=8.08 (2 H, d, *J*=1.9 Hz), 7.48 (1 H, t, *J*=1.9 Hz), 5.71 (2 H, s), 1.36 ppm (9 H, s); ^13^C NMR (125 MHz, CDCl_3_): *δ*=26.2, 43.7, 56.6, 125.3, 130.0, 130.3, 135.7, 163.4, 204.2 ppm; [*M*+H]^+^=313; HRMS C_13_H_14_Cl_2_N_4_O calcd: 313.0586, obsd: 313.0601.

**2-(5-(3,5-Dichlorophenyl)-2*H*-tetrazol-2-yl)-*N***,***N*****-dimethylacetamide (23)**: A mixture of 5-(3,5-dichlorophenyl)tetrazole (0.215 g, 1.0 mmol), NaI (0.150 g, 1.0 mmol) and K_2_CO_3_ (0.210 g, 1.5 mmol) in DMF (5 mL) was heated at 90 °C until effervescence was nearly complete. 2-Chloro-*N*,*N*-dimethylacetamide (0.11 mL, 1.1 mmol) was added, and the mixture was heated at 90 °C overnight. The mixture was diluted with H_2_O (15 mL) and allowed to stand for several hours. The precipitate was collected by filtration and washed with H_2_O and Et_2_O to give the title compound **23** (0.150 g, 50 %) as a white solid: ^1^H NMR (CDCl_3_, 300 K): *δ*=8.09 (2 H, d, *J*=1.9 Hz), 7.47 (1 H, t, *J*=1.9 Hz), 5.58 (2 H, s), 3.17 (3 H, s), 3.07 ppm (3 H, s); [*M*+H]^+^=300; HRMS C_11_H_11_Cl_2_N_5_O calcd: 300.0413, obsd: 300.0411.

Compounds below were prepared according to the same procedures as described above for the preparation of compound **23**, using the corresponding aryl tetrazole and α-haloketone.

**3,3-Dimethyl-1-(5-phenyl-2*H*-tetrazol-2-yl)butan-2-one (14)**: ^1^H NMR (CDCl_3_, 300 K): *δ*=8.17 (2 H, m), 7.51 (3 H, m), 5.70 (2 H, s), 1.35 ppm (9 H, s); [*M*+H]^+^=245; HRMS C_13_H_16_N_4_O calcd: 245.1397, obsd: 245.1387.

**1-(5-(3-Fluorophenyl)-2*H*-tetrazol-2-yl)-3,3-dimethylbutan-2-one (17)**: ^1^H NMR (CDCl_3_, 300 K): *δ*=7.98 (1 H, d, *J*=7.7 Hz), 7.88 (1 H, d, *J*=9.3 Hz), 7.48 (1 H, m), 7.19 (1 H, td, *J*=8.3 and 2.1 Hz), 5.71 (2 H, s), 1.32 ppm (9 H, s); [*M*+H]^+^=263; HRMS C_13_H_15_FN_4_O calcd: 263.1303, obsd: 263.1291.

**1-(5-(3,5-Difluorophenyl)-2*H*-tetrazol-2-yl)-3,3-dimethylbutan-2-one (19)**: ^1^H NMR (CDCl_3_, 300 K): *δ*=7.72 (2 H, m), 6.94 (1 H, tt, *J*=8.8 and 2.4 Hz), 5.71 (2 H, s), 1.36 ppm (9 H, s); [*M*+H]^+^=281; HRMS C13H14F2N4O calcd: 281.1208, obsd: 281.1210.

**1-(5-(3,5-Dichlorophenyl)-2*H*-tetrazol-2-yl)propan-2-one (21**): ^1^H NMR (CDCl_3_, 300 K): *δ*=8.08 (2 H, d, 1.9 Hz), 7.48 (1 H, t, 1.9 Hz), 5.51 (2 H, s), 2.30 ppm (3 H, s); [*M*+H]^+^=271/273; HRMS C_10_H_8_Cl_2_N_4_O calcd: 271.0148, obsd: 271.0141.

**1-(5-(3,5-Dichlorophenyl)-2*H*-tetrazol-2-yl)butan-2-one (22)**: ^1^H NMR (CDCl_3_, 300 K): *δ*=8.09 (2 H, d, 1.9 Hz), 7.50 (1 H, t, 1.9 Hz), 5.52 (2 H, s), 2.57 (2 H, q, *J*=7.2 Hz), 1.19 ppm (3 H, t, *J*=7.2 Hz); [*M*+H]^+^=285/287; HRMS C_11_H_10_Cl_2_N_4_O calcd: 285.0304, obsd: 285.0292.

**2-(5-(3,5-Difluorophenyl)-2*H*-tetrazol-2-yl)-1-phenylethanone (24)**: ^1^H NMR (CDCl_3_, 300 K): *δ*=8.02 (2 H, m), 7.72 (3 H, m), 7.58 (2 H, t, *J*=7.8 Hz), 6.93 (1 H, tt, *J*=8.8 and 2.4 Hz), 6.16 ppm (2 H, s); [*M*+H]^+^=301; HRMS C_11_H_6_F_2_N_4_O calcd: 301.0895, obsd: 301.0901.

Compounds **15**, **16** and **18** were obtained from Maybridge.

### Compounds in series 1 c:

***N*****-(4-Chlorobenzyl)-2-(3-(trifluoromethyl)-4,5,6,7-tetrahydro-1*H*-indazol-1-yl)acetamide (26)**: To a stirred suspension of 2-(trifluoroacetyl)cyclohexanone (1 g, 5.151 mmol) and ethylhydrazinoacetate hydrochloride (2.39 g, 15.5 mmol) in EtOH (30 mL) was added Et_3_N (2.17 mL, 15.5 mmol) and the reaction heated at reflux for 24 h. The reaction mixture was cooled to room temperature and the solvent removed in vacuo. The resultant crude residue was taken up in CH_2_Cl_2_, washed (H_2_O, brine), dried (MgSO_4_) and concentrated in vacuo. The resultant crude residue was purified by column chromatography, eluting with 0–50 % EtOAc/hexane, to give ethyl 2-(3-(trifluoromethyl)-4,5,6,7-tetrahydro-1*H*-indazol-1-yl)acetate (864 mg, 61 %) as a white solid; [*M*+H]^+^=277.

To a stirred solution of ethyl 2-(3-(trifluoromethyl)-4,5,6,7-tetrahydro-1*H*-indazol-1-yl)acetate (691 mg, 2.5 mmol) in 1:1 THF/H_2_O (30 mL) was added 2 m (aq) NaOH (5 mL, 10 mmol), and the reaction stirred for 3 h. The solvent was then removed in vacuo to give a crude residue which was suspended in H_2_O, acidified (1 m aq. HCl), stirred for 5 min, filtered and the precipitate dried in vacuo to give 2-(3-(trifluoromethyl)-4,5,6,7-tetrahydro-1*H*-indazol-1-yl)acetic acid (550 mg, 89 %) as a white solid; [*M*+H]^+^=249.

To a stirred solution of 2-(3-(trifluoromethyl)-4,5,6,7-tetrahydro-1*H*-indazol-1-yl)acetic acid (124 mg, 0.5 mmol), 4-chlorobenzylamine (0.061 mL, 0.5 mmol) and 1-hydroxybenzotriazole hydrate (68 mg, 0.5 mmol) in *N*,*N*-dimethylformamide (DMF; 3 mL) at 50 °C was added *N*-(3-dimethylaminopropyl)-*N*′-ethylcarbodiimide hydrochloride (96 mg, 0.5 mmol) and *N*,*N*′-diisopropylethylamine (0.18 mL, 1 mmol) and the reaction stirred for 16 h. The reaction mixture was cooled to room temperature, taken up in EtOAc, washed (H_2_O, brine), dried (MgSO_4_) and concentrated in vacuo. The resultant crude residue was purified by reversed-phase HPLC (method A), to give title compound **26** (120 mg, 79 %) as a white solid: ^1^H NMR ([D_6_]DMSO, 500 MHz): *δ*=8.78 (1 H, t, *J*=5.9 Hz), 7.41 (2 H, m), 7.30 (2 H, m), 4.84 (2 H, s), 4.30 (2 H, d, *J*=3.0 Hz), 2.56 (2 H, m), 2.51 (2 H, m), 1.74 (2 H, m), 1.67 ppm (2 H, m); ^13^C NMR (125 MHz, CDCl_3_): *δ*=19.9, 21.2, 21.9, 22.2, 42.8, 52.3, 116.1, 120.6, 122.7, 128.8, 128.9, 133.5, 136.0, 140.1, 166.3 ppm; [*M*+H]^+^=372; HRMS C_17_H_17_ClF_3_N_3_O calcd: 372.1085, obsd: 372.1103.

***N***,***N*****-Diethyl-2-(3-(trifluoromethyl)-4,5,6,7-tetrahydro-1*H*-indazol-1-yl)acetamide (35)**: By proceeding in a similar manner to compound **26**, using 2-(3-(trifluoromethyl)-4,5,6,7-tetrahydro-1*H*-indazol-1-yl)acetic acid and diethylamine the title compound **35** (100 mg, 80 %) was obtained as a white solid. ^1^H NMR ([D_6_]DMSO, 500 MHz): *δ*=5.10 (2 H, s), 3.37 (2 H, q, *J*=7.2, 7.5 Hz), 3.27 (2 H, q, *J*=7.1 Hz), 1.71 (4 H, m), 1.17 (3 H, t, *J*=7.1 Hz), 1.03 ppm (3 H, t, *J*=7.1 Hz); [*M*+H]^+^=304; HRMS C_14_H_20_F_3_N_3_O calcd: 304.1631, obsd: 304.1628.

Compounds **25**, **27**, **28**, **31**, **32**, **33** and **34** were obtained from Asinex. Compounds **29** and **30** were obtained from ChemDiv.

### Compounds in series 2 a:

**2-Methyl-*N*-((5-methyl-2-phenyl-2*H*-1,2,3-triazol-4-yl)methyl)propane-2-sulfonamide (42)**: *tert*-Butylsulfinyl chloride (0.18 mL, 1.5 mmol) was added to a solution of (5-methyl-2-phenyl-2*H*-1,2,3-triazol-4-yl)methanamine (0.188 g, 1.0 mmol) and Et_3_N (1.4 mL, 10 mmol) in CH_2_Cl_2_ (10 mL) at 0 °C. The mixture was stirred at 0 °C for 2 h and then quenched with aqueous NaHCO_3_. The phases were separated and the aqueous phase was extracted with CH_2_Cl_2_. The combined organic phases were dried (Na_2_SO_4_) and concentrated. Chromatography on silica (50–100 % EtOAc/PE 40:60) gave 2-methyl-*N*-((5-methyl-2-phenyl-2*H*-1,2,3-triazol-4-yl)methyl)propane-2-sulfinamide as an orange solid (0.240 g, 82 %): ^1^H NMR (CDCl_3_, 300 K): *δ*=8.01 (2 H, d, *J*=8.4 Hz), 7.47 (2 H, t, *J*=7.9 Hz), 7.33 (1 H, t, *J*=7.3 Hz), 4.50 (1 H, dd, *J*=14.1 and 4.7 Hz), 4.36 (1 H, dd, *J*=14.1 and 7.5 Hz), 3.60 (1 H, m), 2.42 (3 H, s), 1.27 ppm (9 H, s); [*M*+H]^+^=293.

A solution of 2-methyl-*N*-((5-methyl-2-phenyl-2*H*-1,2,3-triazol-4-yl)methyl)propane-2-sulfinamide (0.146 g, 0.5 mmol) in CH_2_Cl_2_ (7.5 mL) was treated with *m*CPBA (Aldrich, ≤77 %; 0.225 g, ≤1.0 mmol) and stirred at room temperature for 45 min. The reaction was quenched with 2 m aqueous NaHSO_3_ (10 mL) and saturated aqueous NaHCO_3_ (10 mL). The organic phase was applied to a plug of silica and eluted with CH_2_Cl_2_/EtOAc (0→100 %) to give title compound **42** as a white solid (0.107 g, 69 %): ^1^H NMR (CDCl_3_, 300 K): *δ*=8.01 (2 H, m), 7.49 (2 H, m), 7.34 (1 H, tt, *J*=7.4 and 1.1 Hz), 4.51 (2 H, d, *J*=5.7 Hz), 4.43 (1 H, m), 2.44 (3 H, s), 1.47 ppm (9 H, s); [*M*+H]^+^=309; HRMS C_14_H_20_N_4_O_2_S calcd: 309.1307, obsd: 309.1312.

### Compounds in series 2 a, 2 b and 2 c:

Compounds **36**–**41** were obtained from Maybridge. Compounds **43**, **44**, **45**, **53**, and **54** were obtained from ChemDiv. Compound **46** was obtained from ChemBridge. Compounds **47**–**52** were obtained from Asinex. Compounds **55**–**57** were obtained from Life Chemicals. Compound **58** was obtained from Enamine.

### Compounds in series 3:

**1-(3-(3-Fluorophenyl)-1*H*-indazol-1-yl)-3,3-dimethylbutan-2-one (71)**: The synthesis was previously described elsewhere.^20^

3-Iodo-1*H*-indazole was prepared as reported by Edwards et al;^25^ [*M*+H]^+^=245.

A capped process vial containing 3-iodo-1*H*-indazole (366 mg, 1.5 mmol), 3-fluorophenylboronic acid (252 mg, 1.8 mmol), 2 m (aq) Na_2_CO_3_ (1.12 mL, 2.25 mmol) and *tetrakis*-(triphenylphosphine)palladium(0) in 7:3:2 DME/H_2_O/EtOH (3 mL) was degassed, flooded with argon and irradiated (Biotage Initiator) at 150 °C for 20 min. 3-Fluorophenylboronic acid (126 mg, 0.9 mmol) and 2 m (aq) Na_2_CO_3_ (0.56 mL, 1.12 mmol) were added, the system degassed, flooded with argon and irradiated (Biotage Initiator) at 150 °C for 20 min. The reaction mixture was taken up in EtOAc, washed (H_2_O, 1 m (aq) NaOH, brine), dried (MgSO_4_) and concentrated in vacuo. The resultant crude residue was purified by column chromatography, eluting with 0–50 % EtOAc/hexane, to give 3-(3-fluorophenyl)-1*H*-indazole (196 mg, 62 %) as a white solid.

To a stirred suspension of 60 % NaH (72 mg, 1.79 mmol) in DMF (4 mL) was added 3-(3-fluorophenyl)-1*H*-indazole (95 mg, 0.448 mmol) and the reaction stirred for 10 min. To the reaction mixture 1-bromopinacolone (0.24 mL, 1.79 mmol) was added, the reaction heated at 75 °C and stirring continued for 66 h. The reaction mixture was cooled to room temperature, diluted with EtOAc, washed (H_2_O, brine), dried (MgSO_4_) and concentrated in vacuo. The crude residue was purified by column chromatography, eluting with 0–50 % EtOAc/hexane to give title compound **71** (80 mg, 58 %) was obtained as a white solid: ^1^H NMR ([D_6_]DMSO, 500 MHz): *δ*=8.12 (1 H, d, *J*=4.1 Hz), 7.84 (1 H, m), 7.71 (1 H, m), 7.59 (1 H, m), 7.55 (1 H, m), 7.45 (1 H, m), 7.27 (2 H, m), 5.75 (2 H, s), 1.38 ppm (9 H, s); [*M*+H]^+^=311; HRMS C_19_H_19_FN_2_O calcd: 311.1554, obsd: 311.1555.

By proceeding in a similar manner to compound **71**, using 3-(3-fluorophenyl)-1*H*-indazole (or 3-phenyl-1*H*-indazole) and the corresponding alkylating agent, the following compounds were obtained.

**1-(3-Phenyl-1*H*-indazol-1-yl)-3,3-dimethylbutan-2-one (60)**: ^1^H NMR ([D_6_]DMSO, 500 MHz): *δ*=8.09 (1 H, d, *J*=8.2 Hz), 7.97 (2 H, m), 7.54 (3 H, m), 7.43 (2 H, m), 7.25 (1 H, m), 5.73 (2 H, s), 1.28 ppm (9 H, s); ^13^C NMR (125 MHz, CDCl_3_): *δ*=26.4, 43.5, 53.4, 109.0, 121.2, 121.6, 122.1, 126.7, 127.6, 128.0, 128.8, 133.5, 141.8, 144.9, 207.9 ppm; [*M*+H]^+^=293; HRMS C_19_H_20_N_2_O calcd: 293.1648, obsd: 293.1658.

**3-(3-Fluorophenyl)-1-(pyridin-2-ylmethyl)-1*H*-indazole (75)**: ^1^H NMR ([D_6_]DMSO, 500 MHz): *δ*=8.63 (1 H, d, *J*=4.5 Hz), 8.06 (1 H, d, *J*=8.5 Hz), 7.83 (1 H, m), 7.75 (1 H, m), 7.58 (1 H, m), 7.47 (2 H, m), 7.42 (1 H, m), 7.27 (1 H, m), 7.21 (1 H, m), 7.12 (1 H, m), 6.95 (1 H, d, *J*=8.0 Hz), 5.83 ppm (2 H, s); [*M*+H]^+^=304; HRMS C_19_H_14_FN_3_ calcd: 304.1245, obsd: 304.1258.

**3-(3-Fluorophenyl)-1-(pyridin-3-ylmethyl)-1*H*-indazole (76)**: ^1^H NMR ([D_6_]DMSO, 500 MHz): *δ*=8.63 (1 H, d, *J*=2.0 Hz), 8.49 (1H dd, *J*=1.5, 5.0 Hz), 8.13 (1 H, d, *J*=8.5 Hz), 7.87 (2 H, t, *J*=8.5 Hz), 7.72 (1 H, dt, *J*=2.0, 9.5 Hz), 7.69 (1 H, dt, *J*=2.0, 9.5 Hz), 7.58 (1 H, m), 7.50 (1 H, m), 7.35 (1 H, dd, *J*=4.5, 8.0 Hz), 7.28 (2 H, m), 5.81 ppm (2 H, s); [*M*+H]^+^=304; HRMS C_19_H_14_FN_3_ calcd: 304.1245, obsd: 304.1235.

**4-((3-(3-Fluorophenyl)-1*H*-indazol-1-yl)methyl)-2,5-dimethyloxazole (77)**: ^1^H NMR ([D_6_]DMSO, 500 MHz): *δ*=8.10 (1 H, d, *J*=8.5 Hz), 7.83 (1 H, m), 7.77 (1 H, m), 7.71 (1 H, m), 7.58 (1 H, m), 7.48 (1 H, m), 7.27 (2 H, m), 5.52 (2 H, bs), 3.35 (3 H, bs), 2.36 ppm (3 H, bs); [*M*+H]^+^=322; HRMS C_19_H_16_FN_3_O calcd: 322.1350, obsd: 322.1345.

**3-((3-(3-Fluorophenyl)-1*H*-indazol-1-yl)methyl)-5-phenylisoxazole (78)**: ^1^H NMR ([D_6_]DMSO, 500 MHz): *δ*=8.15 (1 H, d, *J*=8.0 Hz), 7.86 (4 H, m), 7.77 (1 H, m), 7.59 (1 H, m), 7.51 (5 H, m), 7.30 (2 H, m), 6.95 ppm (2 H, s); [*M*+H]^+^=370; HRMS C_23_H_16_FN_3_O calcd: 370.1350, obsd: 370.1345.

**3-((3-(3-Fluorophenyl)-1*H*-indazol-1-yl)methyl)-5-methylisoxazole (79)**: ^1^H NMR ([D_6_]DMSO, 500 MHz): *δ*=8.13 (1 H, d, *J*=8.50), 7.86 (1 H, m), 7.80 (1 H, m), 7.74 (1 H, m), 7.59 (1 H, m), 7.51 (1 H, m), 7.28 (2 H, m), 6.08 (1 H, s), 5.80 (2 H, s), 2.32 ppm (3 H, s); [*M*+H]^+^=308; HRMS C_18_H_14_FN_3_O calcd: 308.1194, obsd: 308.1179.

By proceeding in a similar manner to compound **71**, using the corresponding boronic acid and 1-(3-iodo-1*H*-indazol-1-yl)-3,3-dimethylbutan-2-one, the following compounds were obtained:

**1-(3-(4-Chlorophenyl)-1*H*-indazol-1-yl)-3,3-dimethylbutan-2-one (61)**: ^1^H NMR ([D_6_]DMSO, 500 MHz): *δ*=8.09 (1 H, d, *J*=8.3 Hz), 8.00 (2 H, m), 7.59 (2 H, m), 7.55 (1 H, d, *J*=8.5 Hz), 7.45 (1 H, m), 7.27 (1 H, m), 5.74 (2 H, s), 1.28 ppm (9 H, s); [*M*+H]^+^=327/329; HRMS C_19_H_19_ClN_2_O calcd: 327.1259, obsd: 327.1265.

**1-(3-(1*H*-Indol-4-yl)-1*H*-indazol-1-yl)-3,3-dimethylbutan-2-one (62)**: ^1^H NMR ([D_6_]DMSO, 500 MHz): *δ*=11.21 (1 H, s), 8.00 (1 H, d, *J*=8.2 Hz), 7.55 (2 H, m), 7.44 (3 H, m), 7.24 (2 H, m), 6.88 (1 H, m), 5.75 (2 H, s), 1.30 ppm (9 H, s); [*M*+H]^+^=332; HRMS C_21_H_21_N_3_O calcd: 332.1757, obsd: 332.1754.

**3,3-Dimethyl-1-(3-(1-methyl-1*H*-indol-4-yl)-1*H*-indazol-1-yl)butan-2-one (63)**: ^1^H NMR ([D_6_]DMSO, 500 MHz): *δ*=8.01 (1 H, d, *J*=8.4 Hz), 7.57 (3 H, m), 7.43 (1 H, t, *J*=7.5 Hz), 7.39 (1 H, d, *J*=3.2 Hz), 7.33 (1 H, t, *J*=7.8 Hz), 7.23 (1 H, t, *J*=7.5 Hz), 6.87 (1 H, d, *J*=3.0 Hz), 5.75 (2 H, s), 3.86 (3 H, s), 1.30 ppm (9 H, s); [*M*+H]^+^=346; HRMS C_22_H_23_N_3_O calcd: 346.1914, obsd: 346.1913.

**3,3-Dimethyl-1-(3-(naphthalen-2-yl)-1*H*-indazol-1-yl)butan-2-one (64)**: ^1^H NMR ([D_6_]DMSO, 500 MHz): *δ*=8.56 (1 H, s), 8.30 (1 H, d, *J*=8.4 Hz), 8.14 (2 H, m), 8.06 (1 H, d, *J*=8.6 Hz), 7.97 (1 H, d, 7.7 Hz), 7.57 (3 H, m), 7.47 (1 H, m), 7.31 (1 H, t, *J*=7.5 Hz), 5.78 (2 H, s), 1.29 ppm (9 H, s); [*M*+H]^+^=343; HRMS C_23_H_22_N_2_O calcd: 343.1805, obsd: 343.1806.

**3,3-Dimethyl-1-(3-(pyridin-4-yl)-1*H*-indazol-1-yl)butan-2-one (65)**: ^1^H NMR ([D_6_]DMSO, 500 MHz): *δ*=8.69 (2 H, dd, *J*=1.6, 6.1 Hz), 8.22 (1 H, d, *J*=8.3 Hz), 7.97 (2 H, dd, *J*=1.6, 6.2 Hz), 7.59 (1 H, d, *J*=8.5 Hz), 7.48 (1 H, t, *J*=7.5 Hz), 7.32 (1 H, t, *J*=7.5 Hz), 5.82 (2 H, s), 1.28 ppm (9 H, s); [*M*+H]^+^=294; HRMS C_18_H_19_N_3_O calcd: 294.1601, obsd: 294.1614.

**1-(3-(Furan-2-yl)-1*H*-indazol-1-yl)-3,3-dimethylbutan-2-one (66)**: ^1^H NMR ([D_6_]DMSO, 500 MHz): *δ*=8.10 (1 H, d, *J*=8.2 Hz), 7.87 (1 H, d, *J*=1.7 Hz), 7.52 (1 H, d, *J*=8.5 Hz), 7.56 (2 H, m), 7.44 (1 H, m), 7.26 (1 H, m), 5.72 (2 H, s), 1.27 ppm (9 H, s); [*M*+H]^+^=283; HRMS C_17_H_18_N_2_O_2_ calcd: 283.1441, obsd: 283.1452.

**1-(3-(3-Pyridyl)-1*H*-indazol-1-yl)-3,3-dimethylbutan-2-one (67)**: ^1^H NMR ([D_6_]DMSO, 500 MHz): *δ*=9.17 (1 H, m), 8.63 (1 H, dd, *J*=1.6, 4.8 Hz), 8.35 (1 H, dt, *J*=1.9 7.9 Hz), 8.12 (1 H, d, *J*=8.3 Hz), 7.56 (2 H, m), 7.47 (1 H, m), 7.29 (1 H, m), 5.77 (2 H, s), 1.28 ppm (9 H, s); [*M*+H]^+^=294; HRMS C_18_H_19_N_3_O calcd: 294.1601, obsd: 294.1616.

**3,3-Dimethyl-1-(3-(thiophen-3-yl)-1*H*-indazol-1-yl)butan-2-one (68)**: ^1^H NMR ([D_6_]DMSO, 500 MHz): *δ*=8.13 (2 H, m), 7.69 (2 H, m), 7.49 (1 H, d, *J*=8.5 Hz), 7.42 (1 H, m), 7.25 (1 H, m), 5.70 (2 H, s), 1.27 ppm (9 H, s); [*M*+H]^+^=299; HRMS C_17_H_18_N_2_OS calcd: 299.1213, obsd: 299.1219.

**1-(3-(1-Isobutyl-1*H*-pyrazol-4-yl)-1*H*-indazol-1-yl)-3,3-dimethylbutan-2-one (69)**: ^1^H NMR ([D_6_]DMSO, 500 MHz): *δ*=8.39 (1 H, s), 8.04 (1 H, d, *J*=8.1 Hz), 7.99 (1 H, s), 7.45 (1 H, d, *J*=8.4 Hz), 7.39 (1 H, m), 7.19 (1 H, t, *J*=7.1 Hz), 5.66 (2 H, s), 4.01 (2 H, d, *J*=7.2 Hz), 2.20 (1 H, m), 1.26 (9 H, s), 0.88 ppm (6 H, d, *J*=6.6 Hz); [*M*+H]^+^=339; HRMS C_20_H_26_N_4_O calcd: 339.2179, obsd: 339.2183.

**1-(3-(3-Chlorophenyl)-1*H*-indazol-1-yl)-3,3-dimethylbutan-2-one (70)**: ^1^H NMR ([D_6_]DMSO, 500 MHz): *δ*=8.11 (1 H, d, *J*=8.2 Hz), 7.97 (1 H, m), 7.95 (1 H, m), 7.56 (2 H, m), 7.47 (2 H, m), 7.28 (1 H, t, *J*=7.5 Hz), 5.77 (2 H, s), 1.30 ppm (9 H, s); [*M*+H]^+^=327/329; HRMS C_19_H_19_ClN_2_O calcd: 327.1259, obsd: 327.1262.

Compound **59** was obtained from ChemDiv.

**3-(*tert*-Butylsulfonylmethyl)-1-(3-fluorophenyl)-1*H*-indazole (73)**: To a stirred suspension of indazole-3-carboxylic acid (5 g, 30.8 mmol) in MeOH (125 mL) was added concd H_2_SO_4_ (0.5 mL) and the reaction heated at reflux for 16 h. The reaction mixture was cooled to room temperature and the solvent removed in vacuo. The resultant crude residue was taken up in CH_2_Cl_2_, washed (saturated NaHCO_3_ solution), dried (MgSO_4_) and concentrated in vacuo to give methyl 1*H*-indazole-3-carboxylate (4.95 g, 91 %) as a white solid; [*M*+H]^+^=177.

To a stirred solution of methyl 1*H*-indazole-3-carboxylate (881 mg, 5 mmol) in CH_2_Cl_2_ (125 mL), was added 3-fluorophenylboronic acid (1.40 g, 10 mmol), pyridine (0.81 mL, 10 mmol), copper(II) acetate (1.36 g, 7.5 mmol) and molecular sieves (4 Å, 3.82 g) and the reaction stirred (open to air) for 46 h. The reaction mixture was then filtered through a pad of Celite and concentrated in vacuo to give a crude residue which was purified by column chromatography, eluting with 0–50 % Et_2_O/hexane, to give methyl 1-(3-fluorophenyl)-1*H*-indazole-3-carboxylate (332 mg, 25 %) as a white solid; [*M*+H]^+^=271.

To a stirred solution of methyl 1-(3-fluorophenyl)-1*H*-indazole-3-carboxylate (329 mg, 1.2 mmol) in THF (10 mL) at 0 °C, under an inert atmosphere, was added LiAlH_4_ (185 mg, 4.9 mmol), the reaction warmed to room temperature and stirred for 16 h. The reaction mixture was quenched (Na_2_SO_4_⋅10 H_2_O), filtered through a pad of Celite and concentrated in vacuo. The resultant crude residue was taken up in 3:1 THF/CH_2_Cl_2_ (12 mL), PPh_3_ (479 mg, 1.826 mmol) and *N*-chlorosuccinimide (NCS; 244 mg, 1.8 mmol) added and the reaction stirred for 24 h. Extra PPh_3_ (479 mg, 1.8 mmol) and NCS (244 mg, 1.8 mmol) were then added and stirring continued for 24 h. The reaction mixture was then concentrated in vacuo to give a crude residue which was taken up in EtOAc, washed (saturated NaHCO_3_ solution, H_2_O, and brine), dried (MgSO_4_) and concentrated in vacuo. The resultant crude residue was purified by column chromatography, eluting with 0–50 % Et_2_O/hexane, to give 3-(chloromethyl)-1-(3-fluorophenyl)-1*H*-indazole (143 mg, 45 %) as a straw-coloured oil; [*M*+H]^+^=261/263.

To a stirred solution of 3-(chloromethyl)-1-(3-fluorophenyl)-1*H*-indazole (46 mg, 0.18 mmol) in CH_3_CN (3 mL) was added Cs_2_CO_3_ (63 mg, 0.19 mmol) and *tert*-butylthiol (0.022 mL, 0.19 mmol) and the reaction stirred for 16 h. Extra Cs_2_CO_3_ (32 mg, 0.097 mmol) and *tert*-butylthiol (0.011 mL, 0.097 mmol) were then added and stirring continued for 6 h. The reaction mixture was taken up in CH_2_Cl_2_, washed (H_2_O, brine), dried (MgSO_4_) and concentrated in vacuo. The resultant crude residue was purified by column chromatography, eluting with 0–50 % Et_2_O/hexane, to give 3-(*tert*-butylthiomethyl)-1-(3-fluorophenyl)-1*H*-indazole (45 mg, 81 %) as a colourless oil; [*M*+H]^+^=315.

To a stirred solution of 3-(*tert*-butylthiomethyl)-1-(3-fluorophenyl)-1*H*-indazole (45 mg, 0.14 mmol) in CH_2_Cl_2_ (5 mL) was added 70 % *m*CPBA (141 mg, 0.57 mmol) and the reaction stirred for 2 h. NaHSO_3_ solution (2 m aq) was added, the layers separated, the organic layer washed (saturated NaHCO_3_ solution) and filtered through a pad of silica, eluting with CH_2_Cl_2_ and EtOAc. The filtrate was concentrated in vacuo to give a crude residue which was purified by reversed-phase HPLC (method A), to give title compound **73** (35 mg, 71 %) as a white solid: ^1^H NMR ([D_6_]DMSO, 500 MHz): *δ*=7.97 (1 H, dt, *J*=0.9, 8.1 Hz), 7.95 (1 H, dt, *J*=1.0, 8.5 Hz), 7.67 (3 H, m), 7.57 (1 H, m), 7.36 (1 H, m), 7.30 (1 H, m), 5.00 (2 H, s), 1.44 ppm (9 H, s); [*M*+H]^+^=347; HRMS C_18_H_19_FN_2_O_2_S calcd: 347.1224, obsd: 347.1236.

**3-(*tert*-Butylsulfonylmethyl)-1-phenyl-1*H*-indazole (72)**: By proceeding in a similar manner to 3-(*tert*-butylsulfonylmethyl)-1-(3-fluorophenyl)-1*H*-indazole (see compound **73**), except using phenylboronic acid, title compound **72** (35 mg, 81 %) was obtained as a white solid. ^1^H NMR ([D_6_]DMSO, 500 MHz): *δ*=7.96 (1 H, dt, *J*=0.9, 8.1 Hz), 7.87 (1 H, dt, *J*=0.9, 8.6 Hz), 7.79 (2 H, m), 7.63 (2 H, m), 7.54 (1 H, m), 7.45 (1 H, m), 7.33 (1 H, m), 5.00 (2 H, s), 1.44 ppm (9 H, s); ^13^C NMR (125 MHz, CDCl_3_): *δ*=23.9, 48.0, 61.1, 110.5, 121.9, 122.3, 123.0, 124.8, 127.1, 127.7, 129.5, 135.8, 139.7, 140.1 ppm; [*M*+H]^+^=329; HRMS C_18_H_20_N_2_O_2_S calcd: 329.1318, obsd: 329.1302.

**1-(*tert*-Butylsulfonylmethyl)-3-(3-fluorophenyl)-1*H*-indazole (74)**: 3-(3-Fluorophenyl)-1*H*-indazole (see compound **71**) (0.106 g, 0.5 mmol) was alkylated with *tert*-butyl(chloromethyl)sulfane (0.104 g, 0.75 mmol), as in accordance with reported procedures^28^ to give the intermediate 1-(*tert*-butylthiomethyl)-3-(3-fluorophenyl)-1*H*-indazole (0.055 g, 35 %). ^1^H NMR (CDCl_3_, 300 K) *δ*=8.03 (1 H, dt, *J*=7.5 and 0.8 Hz), 7.79 (1 H, dt, *J*=7.7 and 1.2 Hz), 7.71 (1 H, ddd, *J*=10.0, 2.5 and 1.0 Hz), 7.65 (1 H, dt, *J*=8.5 and 0.8 Hz), 7.51-7.47 (2 H, m), 7.29 (1 H, m), 7.12 (1 H, tdd, *J*=8.5, 2.5 and 0.8 Hz), 5.68 (2 H, s), 1.34 ppm (9 H, s); [*M*+H]^+^=315.

A solution of this sulfide in CH_2_Cl_2_ (2 mL) was treated with *m*CPBA (Aldrich, ≤77 %; 0.11 g, ≤0.5 mmol) and stirred at room temperature for 90 min. The reaction was quenched with 2 m aqueous NaHSO_3_ (10 mL) and saturated aqueous NaHCO_3_ (10 mL). The organic phase was applied to a plug of silica and eluted with CH_2_Cl_2_/EtOAc (0→100 %) to give the title compound **74** as a white solid (0.054 g, 89 %): ^1^H NMR (CDCl_3_, 300 K): *δ*=8.01 (1 H, dt, *J*=8.2 and 0.9 Hz), 7.78 (1 H, dt, *J*=8.5 and 0.8 Hz), 7.76 (1 H, ddd, *J*=11.7, 1.4 and 1.0 Hz), 7.67 (1 H, ddd, *J*=9.9, 2.5 and 1.5 Hz), 7.55 (1 H, ddd, *J*=8.4, 7.0 and 1.0 Hz) 7.50 (1 H, td, *J*=8.1 and 5.9 Hz), 7.34 (1 H, ddd, *J*=8.2, 6.8 and 0.8 Hz), 7.15 (1 H, tdd, *J*=8.4, 2.6 and 0.9 Hz), 5.76 (2 H, s), 1.43 ppm (9 H, s); ^13^C NMR (125 MHz, CDCl_3_): *δ*=23.4, 61.2, 64.6, 110.0, 114.4, 115.5, 121.1, 122.3, 123.2, 127.9, 130.5, 134.8, 142.2, 145.1, 161.2, 164.1 ppm; [*M*+H]^+^=347; HRMS C_18_H_19_FN_2_O_2_S calcd: 347.1224, obsd: 347.1214.

**2-(3-(3-Fluorophenyl)-1*H*-indazol-1-yl)-*N*-(3-(4-methylpiperazin-1-yl)propyl)acetamide (80)**: To a stirred suspension of 60 % NaH (754 mg, 18.9 mmol) in DMF (20 mL) under an inert atmosphere, was added 3-(3-fluorophenyl)-1*H*-indazole (1.0 g, 4.7 mmol) in DMF (10 mL) and the reaction was stirred for 15 min. To the reaction mixture was added ethyl bromoacetate (2.09 mL, 18.9 mmol), the reaction was warmed to 75 °C and stirring continued for 66 h. The reaction mixture was cooled to room temperature, taken up in EtOAc, washed (H_2_O, brine), dried (MgSO_4_) and concentrated in vacuo. The crude residue was taken up in 1:1 THF/H_2_O (30 mL) and 2 m (aq) NaOH (9.43 mL, 18.9 mmol), the reaction was heated at 50 °C and stirred for 3 h. The reaction was cooled to room temperature, diluted with H_2_O, extracted (EtOAc), the aqueous layer acidified (1 m (aq) HCl, pH 2), extracted (EtOAc). The organic layer was dried (MgSO_4_) and concentrated in vacuo to give 2-(3-(3-fluorophenyl)-1*H*-indazol-1-yl)acetic acid (980 mg, 77 %) as a white foam; [*M*+H]^+^=471.

By proceeding in a similar manner to **26**, except using 2-(3-(3-fluorophenyl)-1*H*-indazol-1-yl)acetic acid and 1-(2-aminopropyl)-4-methylpiperazine, the title compound **80** (65 mg, 43 %) was obtained as a white solid: ^1^H NMR ([D_6_]DMSO, 500 MHz): *δ*=8.25 (1 H, t, *J*=5.4 Hz), 8.11 (1 H, d, *J*=8.4 Hz), 7.85 (1 H, d, *J*=7.9 Hz), 7.72 (1 H, m), 7.68 (1 H, d, *J*=8.9 Hz), 7.58 (1 H, m), 7.47 (1 H, t, *J*=7.7 Hz), 7.27 (2 H, m), 5.16 (2 H, s), 3.12 (2 H, m), 2.51 (8 H, m), 2.27 (2 H, m), 2.12 (3 H, s), 1.58 ppm (2 H, m); [*M*+H]^+^=410; HRMS C_23_H_28_FN_5_O calcd: 410.2351, obsd: 410.2363.

By proceeding in a similar manner to **26**, except using 2-(3-(3-fluorophenyl)-1*H*-indazol-1-yl)acetic acid and the appropriate amine, the following compounds were prepared:

***N*****-(3-(1*H*-Imidazol-1-yl)propyl)-2-(3-(3-fluorophenyl)-1*H*-indazol-1-yl)acetamide (81)**: ^1^H NMR ([D_6_]DMSO, 500 MHz): *δ*=8.41 (1 H, t, *J*=5.4 Hz), 8.25 (1 H, s), 8.12 (1 H, d, *J*=8.3 Hz), 7.84 (1 H, d, *J*=7.9 Hz), 7.72 (2 H, m), 7.58 (1 H, m), 7.48 (1 H, t, *J*=7.7 Hz), 7.44 (1 H, m), 7.27 (2 H, m), 7.23 (1 H, m), 5.20 (2 H, s), 4.10 (2 H, m), 3.09 (2 H, m), 1.93 ppm (2 H, m); [*M*+H]^+^=378; HRMS C_21_H_20_FN_5_O calcd: 378.1725, obsd: 378.1739.

***tert*****-Butyl-4-(2-(2-(3-(3-fluorophenyl)-1*H*-indazol-1-yl)acetamido)ethyl)piperazine-1-carboxylate (82)**: ^1^H NMR ([D_6_]DMSO, 500 MHz) *δ* mixture of rotamers *δ*=8.12 (2 H, m), 7.86 (1 H, d, *J*=7.6 Hz), 7.72 (2 H, m), 7.59 (1 H, m), 7.48 (1 H, m), 7.28 (2 H, m), 5.22 (2 H, s), 3.20 (2 H, m), 2.51 (8 H, m), 2.37 (2 H, m), 1.40(5 H, s), 1.39 ppm (4 H, s); [*M*+H]^+^=482; HRMS C_26_H_32_FN_5_O_3_ calcd: 482.2562, obsd: 482.2570.

***N*****-(2-(Dimethylamino)ethyl)-2-(3-(3-fluorophenyl)-1*H*-indazol-1-yl)acetamide (83)**: NMR ^1^H NMR ([D_6_]DMSO, 500 MHz): *δ*=8.22 (1 H, t, *J*=5.5 Hz), 8.12 (1 H, d, *J*=8.2 Hz), 7.85 (1 H, d, *J*=7.9 Hz), 7.72 (1 H, m), 7.69 (1 H, d, *J*=8.5 Hz), 7.59 (1 H, m), 7.47 (1 H, m), 7.26 (2 H, m), 5.19 (2 H, s), 3.20 (2 H, m), 2.31 (2 H, m), 2.15 ppm (6 H, s); [*M*+H]^+^=341; HRMS C_19_H_21_FN_4_O calcd: 341.1772, obsd: 341.1767.

***N*****-(3-(Dimethylamino)propyl)-2-(3-(3-fluorophenyl)-1*H*-indazol-1-yl)acetamide (84)**: ^1^H NMR ([D_6_]DMSO, 500 MHz): *δ*=8.26 (1 H, t, *J*=5.3 Hz), 8.13 (1 H, d, *J*=8.1 Hz), 7.85 (1 H, d, *J*=7.8 Hz), 7.71 (1 H, m), 7.68 (1 H, d, *J*=8.6 Hz), 7.59 (1 H, m), 7.48 (1 H, m), 7.26 (2 H, m), 5.16 (2 H, s), 3.13 (2 H, m), 2.20 (2 H, m), 2.01 (6 H, s), 1.54 ppm (2 H, m); ^13^C NMR (125 MHz, CDCl_3_): *δ*=24.4, 40.7, 44.5, 52.6, 59.4, 109.5, 114.2, 115.1, 121.2, 122.1, 123.0, 127.3, 130.5, 135.3, 141.7, 144.0, 162.2, 164.2, 167.3 ppm; [*M*+H]^+^=355; HRMS C_20_H_23_FN_4_O calcd: 355.1856, obsd: 355.1862.

***N*****-Methyl-(3-(dimethylamino)propyl)-2-(3-(3-fluorophenyl)-1*H*-indazol-1-yl)acetamide (85)**: ^1^H NMR ([D_6_]DMSO, 500 MHz) mixture of rotamers; *δ*=8.12 (1 H, d, *J*=8.4 Hz), 7.85 (1 H, d, *J*=7.9 Hz), 7.71 (1 H, m), 7.60(2 H, m), 7.44 (1 H, m), 7.25 (2 H, m), 5.60 (1 H, s), 5.50 (1 H, s), 3.48 (1 H, m), 3.30 (1 H, m), 3.14 (1.5 H, s), 2.84 (1.5 H, s), 2.27 (1 H, m), 2.20 (3 H, s), 2.16 (1 H, m), 2.09 (3 H, s), 1.77 (1 H, m), 1.60 ppm (1 H, m); [*M*+H]^+^=369; HRMS C_21_H_25_FN_4_O calcd: 369.2085, obsd: 369.2097.

***N*****-(4-Chlorobenzyl)-2-(3-(3-fluorophenyl)-1*H*-indazol-1-yl)acetamide (86)**: ^1^H NMR ([D_6_]DMSO, 500 MHz): *δ*=8.78 (1 H, t, *J*=5.7 Hz), 8.12 (1 H, d, *J*=8.1 Hz), 7.85 (1 H, d, *J*=7.8 Hz), 7.71 (2 H, m), 7.59 (1 H, m), 7.48 (1 H, t, *J*=7.4 Hz), 7.38 (2 H, d, *J*=8.2 Hz), 7.32 (2 H, d, *J*=8.3 Hz), 7.28 (2 H, m), 5.26 (2 H, s), 4.32 ppm (2 H, d, *J*=6.0 Hz); [*M*+H]^+^=394; HRMS C_22_H_17_ClFN_3_O calcd: 394.1117, obsd: 394.1111.

**2-(3-(3-Fluorophenyl)-1*H*-indazol-1-yl)-*N*-(2-(4-methylpiperazin-1-yl)ethyl)acetamide (90)**: ^1^H NMR ([D_6_]DMSO, 500 MHz): *δ*=8.12 (1 H, d, *J*=8.1 Hz), 8.02 (1 H, t, *J*=5.4 Hz), 7.86 (1 H, d, *J*=8.1 Hz), 7.72 (1 H, m), 7.71 (1 H, d, *J*=8.6 Hz), 7.59 (1 H, m), 7.47 (1 H, t, *J*=7.8 Hz), 7.27 (2 H, m), 5.19 (2 H, s), 3.20 (2 H, m), 2.51 (8 H, m), 2.34 (2 H, m), 2.10 ppm (3 H, s); [*M*+H]^+^=396; HRMS C_22_H_26_FN_5_O calcd: 396.2194, obsd: 396.2212.

***N***,***N*****-Diethyl-2-(3-(3-fluorophenyl)-1*H*-indazol-1-yl)acetamide (87)**: 3-(3-Fluorophenyl)-1*H*-indazole (0.106 g, 0.5 mmol), prepared as in **71**, was alkylated with 2-chloro-*N*,*N*-diethylacetamide (0.102 mL, 0.74 mmol) to give the title compound **87** (0.073 g, 45 %) as a white solid: ^1^H NMR (CDCl_3_, 300 K): *δ*=8.02 (1 H, d, *J*=8.2 Hz), 7.78 (1 H, d, *J*=7.8 Hz), 7.70 (1 H, ddd, *J*=10.1 2.2 and 1.5 Hz), 7.53 (1 H, d, *J*=8.5 Hz), 7.50-7.45 (2 H, m), 7.27 (1 H, m), 7.11 (1 H, td, *J*=7.5 and 2.0 Hz), 5.30 (2 H, s), 3.54 (2 H, q, *J*=7.1 Hz), 3.43 (2 H, q, *J*=7.1 Hz), 1.17 ppm (6 H, m); [*M*+H]^+^=326; HRMS C_19_H_20_FN_3_O calcd: 326.1663, obsd: 326.1663.

**2-(3-(3-Fluorophenyl)-1*H*-indazol-1-yl)-*N***,***N*****-dimethylacetamide (88)**: 3-(3-Fluorophenyl)-1*H*-indazole (0.106 g, 0.5 mmol), prepared as in **71**, was alkylated with 2-chloro-*N*,*N*-dimethylacetamide (0.191 g, 1.6 mmol) to give the title compound **88** (0.114 g, 77 %) as a white solid. ^1^H NMR (CDCl_3_, 300 K): *δ*=1 H, d, *J*=8.2 Hz), 7.78 (1 H, d, *J*=7.7 Hz), 7.70 (1 H, ddd, *J*=10.1, 2.4 and 1.6 Hz), 7.52-7.45 (3 H, m), 7.27 (1 H, m), 7.10 (1 H, ddt, *J*=8.5, 2.6 and 0.8 Hz), 5.32 (2 H, s), 3.18 (3 H, s), 3.01 ppm (3 H, s); [*M*+H]^+^=298; HRMS C_17_H_16_FN_3_O calcd: 298.1350, obsd: 298.1348.

**2-(3-(3-Fluorophenyl)-1*H*-indazol-1-yl)-1-(piperidin-1-yl)ethanone (89)**: Synthesis previously described.^20^ 3-(3-Fluorophenyl)-1*H*-indazole (0.106 g, 0.50 mmol), prepared as in **71**, was alkylated with 2-chloro-1-piperidin-1-ylethanone (0.350 g, 2.2 mmol) to give the title compound (0.05 g, 30 %) as a white solid: ^1^H NMR (CDCl_3_, 300 K): *δ*=8.03 (1 H, d, *J*=8.2 Hz), 7.77 (1 H, d, *J*=7.8 Hz), 7.69 (1 H, dt, *J*=10.0 and 1.7 Hz), 7.55 (1 H, d, *J*=8.5 Hz), 7.50-7.45 (2 H, m), 7.27 (1 H, m), 7.11 (1 H, td, *J*=8.4 and 1.4 Hz), 5.32 (2 H, s), 3.60 (4 H, m), 1.64 (2 H, m), 1.54 (2 H, m), 1.48 ppm (2 H, m); [*M*+H]^+^=338; HRMS C_20_H_20_FN_3_O calcd: 338.1663, obsd: 338.1660.

### Biological assays

*Trypanothione synthetase assay*: The TryS enzyme assays were conducted as reported.^20^

*Cell-based assays*: Proliferation assays using bloodstream form *T. brucei* and human MRC5 fibroblasts were conducted as reported.^17^

### Abbreviations

Glutathione (GSH); human African trypanosomiasis (HAT); spermidine (Spd); single knockout (SKO); Trypanothione synthetase (TryS).
